# Introduction of robotics into a well-established navigation OR team for TKA does not increase surgical time. A one center evaluation

**DOI:** 10.1007/s11701-026-03560-w

**Published:** 2026-06-19

**Authors:** Lorenzo Maggi, Yves Vanderschelden, David Burlot, Nicola Secciani, Benedetto Allotta

**Affiliations:** 1 Department of Industrial Engineering, University of Florence, Via di Santa Marta, 3, 50139 Florence, Italy; 2 Clinique de la Côte d’Émeraude, Saint-Malo, France

**Keywords:** Total knee arthroplasty, Robotic-assisted surgery, Computer navigation, Learning curve, Operative efficiency.

## Abstract

Robotic-assisted Total Knee Arthroplasty (RA-TKA) enhances precision but is historically associated with increased operative times and workflow disruption. This study evaluates whether integrating a novel open-platform robotic effector into an established, familiar navigation workflow mitigates the learning curve and maintains total operative time compared to standard navigation. A retrospective comparative analysis was performed on 236 primary TKAs (142 NAV-TKA vs. 94 RA-TKA) performed by a single high-volume surgical team. Operative times were segmented into five phases using synchronized system logs. Educational cases were excluded. The learning curve was analyzed using Cumulative Sum (CUSUM) control charts. Multi-way analysis across independent cohorts showed a significant global variation in total skin-to-skin time (*p* < 0.001). However, post-hoc pairwise testing demonstrated that while the initial learning phase was significantly longer (Median: 84.00 min), the steady-state proficiency phase (Median: 77.00 min) achieved a comparable time profile to the legacy navigation workflow (Median: 81.00 min; *p* > 0.017), avoiding an overall sustained time penalty. CUSUM analysis identified a learning curve of 58 cases. In the proficiency phase, the active robotic resection time was significantly faster than manual navigated resection (17.95 vs. 19.08 min; *p* = 0.009). The open-platform robotic system successfully integrated into the clinical workflow without introducing an overall sustained time penalty. Retaining a familiar interface effectively cushions the initial efficiency loss typical of closed platforms. Once proficiency is attained, active robotic assistance significantly enhances mechanical resection speed, offsetting the mandatory intra-operative planning time investment.

## Introduction

Total Knee Arthroplasty (TKA) has undergone a paradigm shift over the last two decades, evolving from conventional mechanical instrumentation to sophisticated computer-assisted technologies [1]. While computer-assisted navigation (NAV-TKA) established the principles of digital precision, the adoption of robotic-assisted TKA (RA-TKA) has accelerated dramatically, showing exponential nationwide growth over the last decade [2, 3]. The primary driver for this technological shift is the pursuit of surgical precision; the literature consistently demonstrates that RA-TKA reduces radiographic outliers, improves implant positioning accuracy, and maintains an excellent safety profile without increasing complication rates compared to manual or navigated techniques [4–8]. Some centers have even reported superior patient-reported outcome measures (PROMs) and mid-term survival for robotic cohorts [9].

However, the translation of radiographic accuracy into clinical and institutional efficiency remains a subject of intense debate [10, 11]. A major barrier to widespread robotic adoption is the associated “time penalty” and economic burden. Meta-analyses and high-volume center reports indicate that robotic procedures routinely increase procedural duration and overall operating room occupancy times [12, 13]. This procedural inefficiency is largely driven by the steep learning curve required to master complex proprietary platforms, with reported learning phases ranging from 15 to over 60 cases depending on the system architecture and surgeon experience [14–16]. Although some newer imageless systems have demonstrated shorter learning curves (approximately 7–11 cases) [17–19], the cognitive load associated with learning a completely novel user interface often results in significant temporary workflow disruption [20, 21]. Overcoming this efficiency gap is a critical necessity to ensure equitable patient access to robotic precision without burdening public healthcare resources.

The aim of this study is to compare the operative times of NAV-TKA versus RA-TKA performed by a single surgical team using a novel open-platform and imageless robotic system (ROBIN, Orthokey Italia s.r.l., Italy), designed to integrate directly into the team’s legacy navigation system (BLU-IGS, Orthokey Italia s.r.l., Italy). We hypothesized that, due to the complete retention of the established navigation interface and operational workflow, the introduction of the robotic component would not result in a clinically significant increase in total operative time compared to standard navigation, effectively bypassing the historical initial efficiency penalty.

## Methods

### Study design and patient selection

This retrospective comparative study analyzed data from patients undergoing primary Total Knee Arthroplasty (TKA) at a single high-volume institution (Clinique de la Côte d’Émeraude, Saint-Malo, France). All surgical procedures across both cohorts were performed by a single senior joint arthroplasty surgeon (Y.V.) with an annual volume of approximately 150–200 total knee arthroplasties.

To eliminate procedural confounding variables, the operating room team was standardized, consistently comprising the primary senior surgeon, a dedicated first assistant, and a specialized scrub nurse formally trained in both computer navigation and the specific robotic platform interface. The control cohort consisted of 143 consecutive cases performed between January 2024 and September 2024 using a standard imageless optical navigation system (NAV-TKA group; BLU-IGS, Orthokey Italia s.r.l., Italy), considered completely mastered by the surgical team. The prospective integration of the robotic effector occurred after this period; thus, the investigative cohort included 114 consecutive cases performed between March 2025 and October 2025 using a novel open-platform robotic system (RA-TKA group; ROBIN, Orthokey Italia s.r.l., Italy) integrated into the identical navigation software interface used in the control group. Because the historical control data collection commenced in January 2024, institutional workflows and operating room logistics were entirely unaffected by COVID-19 pandemic disruptions.

For both cohorts, surgical execution followed a standardized protocol: a standard lateral approach was utilized in 100% of cases, and a Functional Alignment philosophy was strictly adopted. Rather than aiming for systematic mechanical neutrality, intra-operative computer navigation planning were leveraged to dynamically accommodate the patient’s individual soft-tissue laxity and constitutional anatomy. Bone resections were adjusted in real-time based on ligamentous tension, substantially minimizing the need for extensive, time-consuming soft-tissue releases. This functional workflow protocol remained identical between the NAV-TKA and RA-TKA groups.

Strict exclusion criteria were applied to ensure a homogeneous comparison of baseline surgical difficulty. Revision surgeries and cases with incomplete automated system log data preventing precise timestamp synchronization were excluded. Crucially, since the institutional protocol indicated Cruciate-Retaining (CR) implants (UOC U2 CR, United Orthopedic Corporation, Taiwan) exclusively for primary cases with mild-to-moderate constitutional deformities, patients presenting with fixed flexion deformities > 15° or severe uncorrectable extra-articular coronal malalignments were excluded from both cohorts, ensuring equivalent intra-operative technical difficulty.

**Fig. 1:**
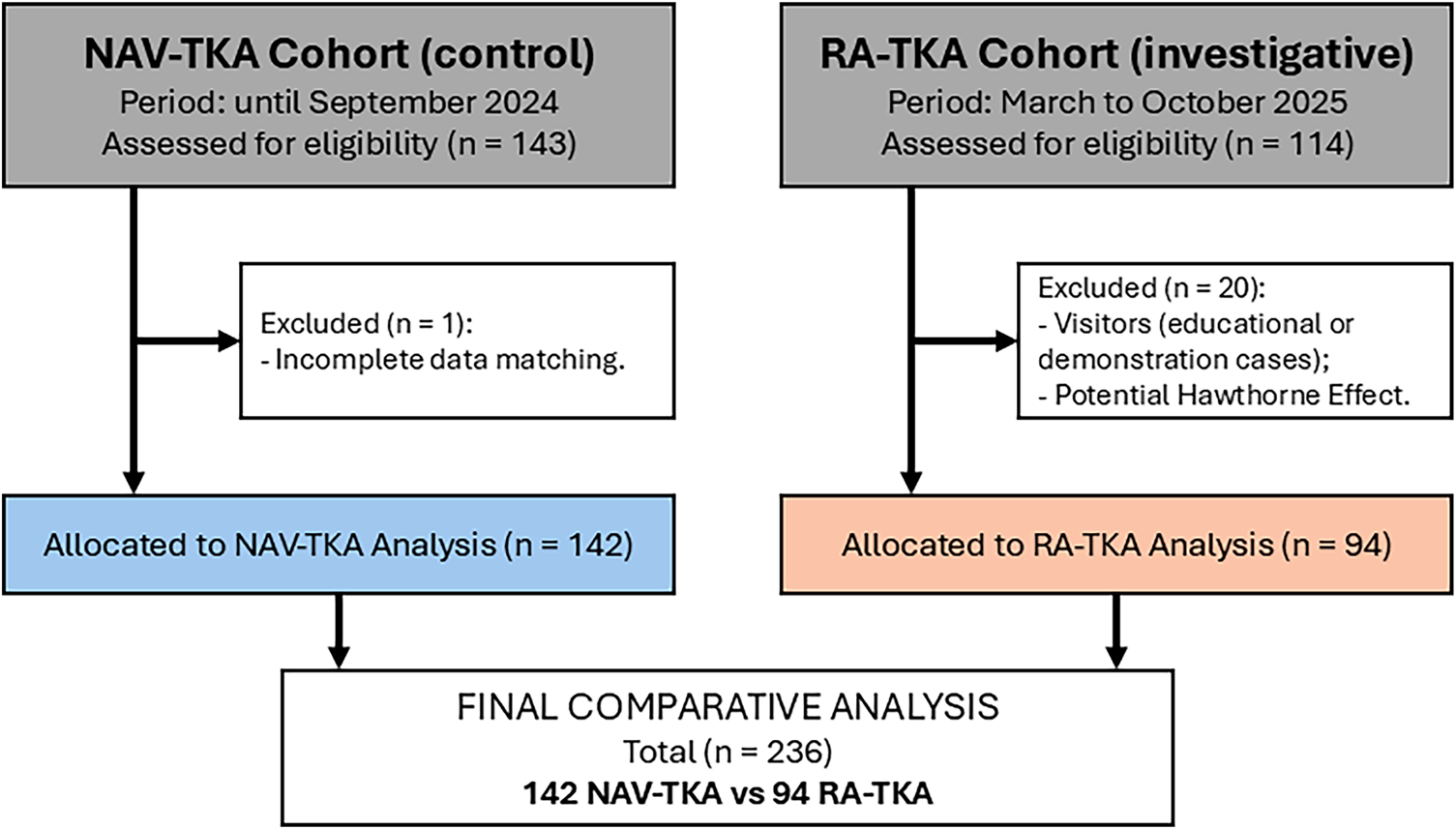
Patient Selection Flowchart

Furthermore, to isolate the intrinsic technical performance of the robotic workflow from educational or external disruptions, cases performed in the presence of external visiting surgeons were excluded (*n* = 20). This was critical to eliminate “demonstration time” or educational pauses, which artificially inflate operative times and do not reflect the system’s true clinical workflow [17]. After applying these criteria, the final comparative analysis included 142 NAV-TKA cases and 94 RA-TKA cases. The patient selection process is detailed in the patient selection flow diagram Fig. 1.

### Data collection and workflow segmentation

To ensure objective assessment of operative efficiency and minimize manual recording bias, operative timestamps were retrieved using a dual-source methodology:

1. **System Logs**: Automated timestamps were extracted from the log files of the navigation unit (shared by both NAV and RA systems) to determine the precise start and end times of computer-assisted phases.
2. **Surgical Records**: “Skin-to-skin” times (from initial incision to final suture completion) were retrieved from the institutional electronic medical records (EMR).

**Fig. 2:**
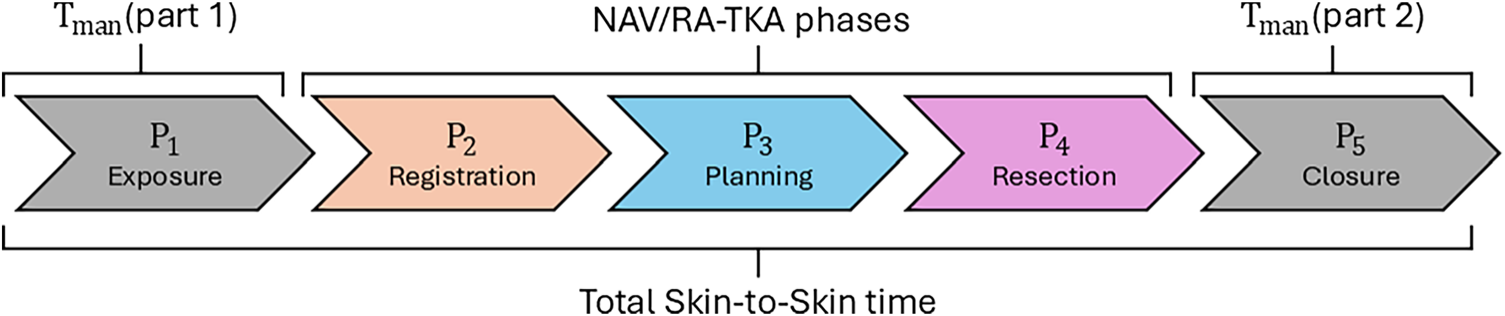
Schematic representation of operative workflow segmentation. The total skin-to-skin timeline is subdivided into five distinct phases (P_1_ – P_5_) based on synchronized timestamps from automated system logs and manual surgical records. P_1_ (Exposure) and P_5_ (Closure) represent the “Manual Phase” (T_man_, performed without direct computer assistance. P_2_ (Registration), P_3_ (Planning), and P_4_ (Resection) represent the “NAV/RA-TKA phases”, actively involving the navigation or robotic interface

System logs were synchronized with surgical records to segment the total operative timeline into five distinct procedural phases (P_1_-P_5_), as illustrated in Fig. 2.

The phases were defined as follows:

- **P**_**1**_ **(Exposure)**: Interval from skin incision to the initiation of the first anatomical landmark acquisition.
- **P**_**2**_ **(Registration)**: Interval from the start of landmark acquisition to the completion of registration and transition to the planning screen.
- **P**_**3**_ **(Planning)**: Duration of intra-operative surgical planning interface usage, ending with the validation of the plan.
- **P**_**4**_ **(Execution/Resection)**: The bone resection phase. In the NAV-TKA group, this represents manual cutting guide position assisted by navigation system and bone sawing. In the RA-TKA group, this represents the bone sawing assisted by robotic unit.
- **P**_**5**_ **(Closure)**: Interval from the completion of bone cuts to final skin suture, including trial reduction and implantation.

### Outcome measures

To evaluate the specific impact of robotic integration on different aspects of the workflow, four distinct time intervals were calculated from the segmented phases:

- **Registration Time (T**_**reg**_**)**: Duration of P_2_.
- **Planning Time (T**_**plan**_**)**: Duration of P_3_.
- **Resection Time (T**_**cut**_**)**: Duration of P_4_.
- **Manual Phase Time (T**_**man**_**)**: The aggregate sum of non-computer-assisted phases (P_1_ + P_5_). This metric serves as an internal control to assess variations in the surgical workflow independent of the navigation/robotic system component.
- **Total Skin-to-Skin Time**: The sum of all phases (P_1_ through P_5_).

### Statistical analysis

Data were analysed using MATLAB (MathWorks, Natick, MA, USA). The normality of data distribution for operative times was assessed using the Lilliefors test. The majority of distributions were found to be non-normal (*p* < 0.05). This right-skewed, log-normal pattern is mathematically expected in surgical safety and efficiency workflows; operative times are naturally bounded by a physical minimum but can extend asymmetric tails driven by the initial learning curve phases and initial software adaptation before workflow stabilization occurs. Consequently, descriptive statistics are reported as Median with Interquartile Range [IQR] (25th–75th percentiles). To analyze the learning curve effect within the robotic cohort, a Cumulative Sum (CUSUM) control chart analysis was performed on the total skin-to-skin times relative to the robotic group mean. The proficiency phase was calculated by identifying the maximum peak (inflection point) of the CUSUM learning curve; this point marks the mathematical transition where the operational trend permanently shifts from a positive slope (procedural durations longer than the global average) to a consistent negative trend (procedural durations shorter than the average), indicating workflow stabilization. This inflection point was utilized to empirically stratify the robotic cohort into an initial “Learning Phase” and a subsequent steady-state “Proficiency Phase”. For inferential hypothesis testing, a multi-way non-parametric Kruskal-Wallis H test was implemented to evaluate global operative time variations across cohorts. To strictly satisfy the mathematical assumption of independent observations and guarantee the unique composition of each group, the multi-way comparison was executed exclusively across the three mutually exclusive cohorts: NAV-TKA (*n* = 142), RA-TKA Learning phase (*n* = 58), and RA-TKA Proficiency phase (*n* = 36). The overall, unstratified robotic cohort (RA-TKA All, *n* = 94) was maintained in the descriptive tables solely for clinical transparency but was omitted from the multi-way inferential calculations. In the presence of a globally significant Kruskal-Wallis result (*p* < 0.05), Dunn’s post-hoc test with Bonferroni correction was applied for pairwise comparisons, utilizing a strict adjusted significance threshold of α = 0.017. Categorical demographic variables were compared using the Chi-Square (χ^2^) test. Statistical significance for all global tests was defined as *p* < 0.05.

## Results

### Normality analysis and demographics

Preliminary statistical assessment using the Lilliefors test confirmed a non-normal distribution for most operative time variables, exhibiting a log-normal pattern. After applying the strict exclusion criteria, a total of 236 procedures were included in the final comparative analysis: 142 in the conventional navigation group (NAV-TKA) and 94 in the robotic group (RA-TKA). Patient demographics and baseline clinical characteristics for both cohorts are summarized in Table 1. The two groups were comparable and homogeneous regarding body mass index (BMI, *p* = 0.295), height (*p* = 0.993), and gender distribution (*p* = 0.544). While patients in the robotic cohort were slightly older (mean 74.0 vs. 71.2 years; *p* = 0.002) and presented a slightly lower body weight (*p* = 0.036), the mean pre-operative hip-knee-ankle (HKA) angle, which represents the primary anatomical determinant of baseline surgical difficulty, was statistically indistinguishable between the NAV-TKA and RA-TKA groups (3.6° ± 4.7° vs. 3.3° ± 5.3°, respectively; *p* = 0.649). Furthermore, all procedures utilized Cruciate-Retaining (CR) implants and a standardized lateral approach performed by the same senior surgeon, ensuring that intra-operative technical complexity was balanced across both cohorts.

**Table 1:** Patient demographics and baseline clinical characteristics

Parameter	NAV-TKA group (*n* = 142)	RA-TKA group (*n* = 94)	*p*-value	Test applied
Age (years, mean ± SD)	**71.2 ± 6.2**	**74.0 ± 7.5**	0.002*	Mann-Whitney U test
Sex (Male/Female, n [%])	60 (42.3%)/ 82 (57.7%)	36 (38.3%)/ 58 (61.7%)	0.544	Chi-Square (χ2) test
BMI (kg/m2, mean ± SD)	29.9 ± 6.9	29.0 ± 5.7	0.295	Mann-Whitney U test
Height (cm, mean ± SD)	166.0 ± 8.9	165.0 ± 9.7	0.993	Mann-Whitney U test
Weight (kg, mean ± SD)	**84.3 ± 17.6**	**79.6 ± 15.4**	0.036*	Mann-Whitney U test
Pre-operative HKA (∘, mean ± SD)	3.6 ± 4.7	3.3 ± 5.3	0.649	Mann-Whitney U test
Surgical Approach	Lateral	Lateral	N/A	Fixed by protocol
Alignment	Functional Alignment (100%)	Functional Alignment (100%)	N/A	Fixed by protocol
Implant Design	UOC U2 CR	UOC U2 CR	N/A	Fixed by protocol
Primary Surgeon	Single Senior (Y.V.)	Single Senior (Y.V.)	N/A	Fixed by protocol

SD: Standard Deviation; BMI: Body Mass Index; HKA: Hip-Knee-Ankle angle; CR: Cruciate-Retaining. *Indicates a statistically significant difference (p < 0.05)

### Operative time comparison (Overall cohort)

**Fig. 3:**
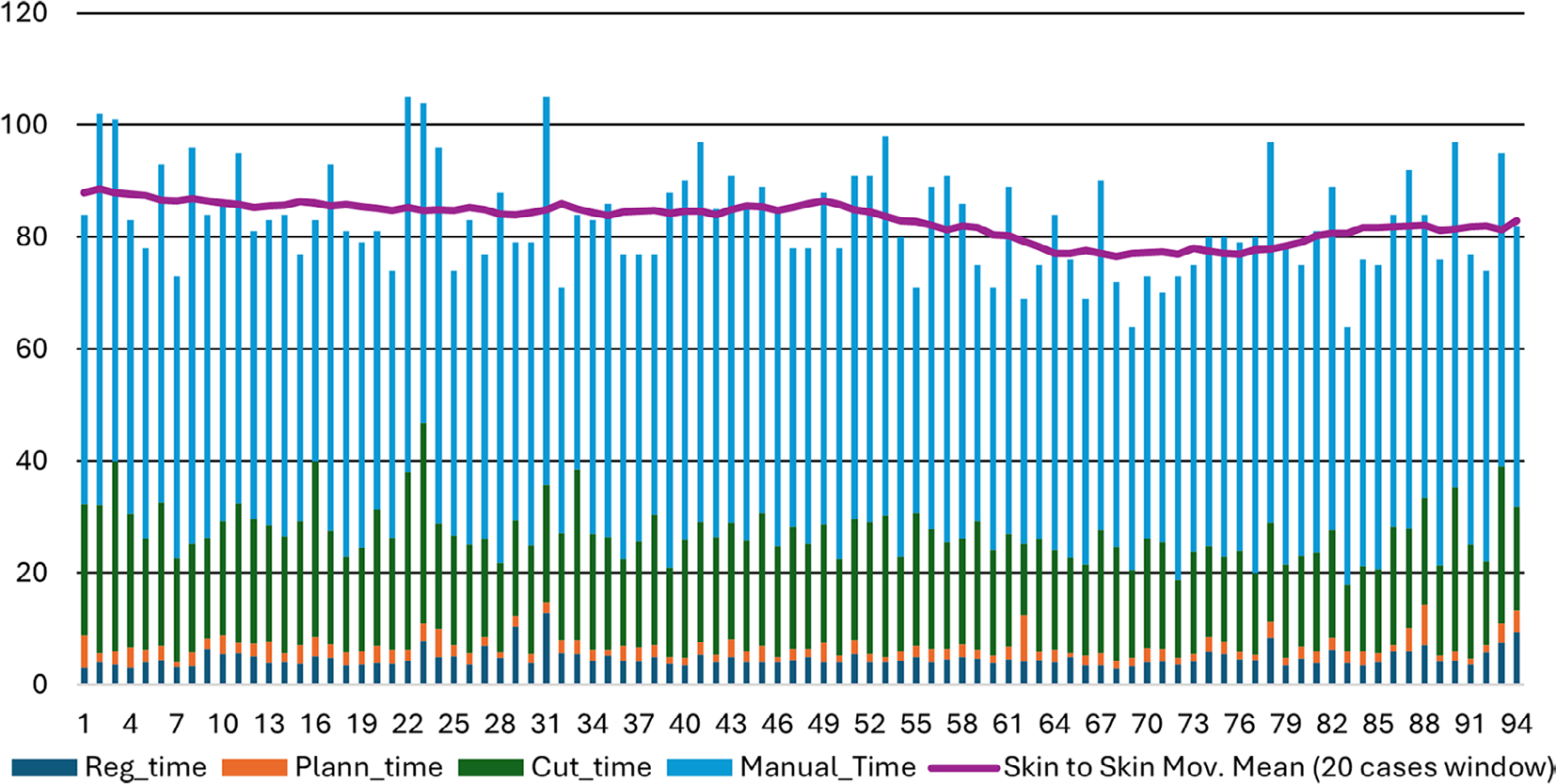
Chronological breakdown of operative time composition for the RA-TKA cohort (*n* = 94). The “Manual Phase” constitutes most of the procedural time, while system-dependent phases remain consistent. The violet line represents the moving average (20-case window) of the total skin-to-skin time, demonstrating workflow stability

**Fig. 4:**
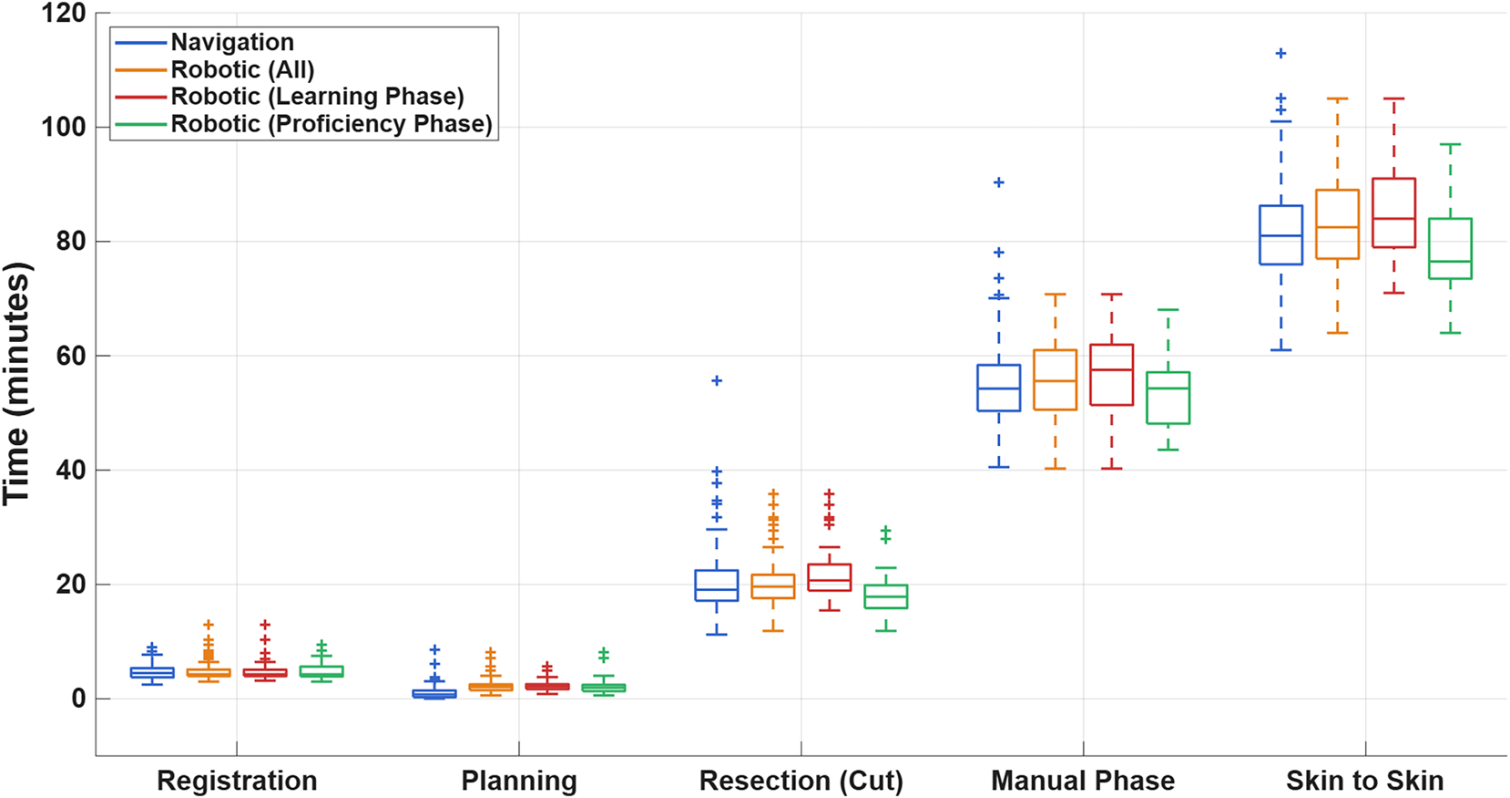
Box-Plot of operative times for each phase and total Skin-to-Skin time, comparison between Navigation cases, overall Robotic cases (RA-TKA All), RA-TKA Learning phase, and RA-TKA Proficiency phase

A multi-way non-parametric comparison was executed across the three independent, mutually exclusive cohorts (NAV-TKA, RA-TKA Learning phase, and RA-TKA Proficiency phase) to assess phase-specific workflow variations. The overall unstratified robotic group (RA-TKA All) is presented alongside the cohorts for baseline descriptive completeness. All synchronized phase durations and global statistical outputs are detailed in Table 2, with the overall distribution summarized in Figs. 3 and 4. Regarding overall surgical efficiency, total skin-to-skin time (T_s2s_) demonstrated a significant global variation across the groups (*p* < 0.001, χ^2^ = 15.10). Dunn’s post-hoc pairwise comparison with Bonferroni correction (α = 0.017) demonstrated that the initial robotic Learning phase (Median: 84.00 min) was significantly longer than both the conventional NAV-TKA cohort (Median: 81.00 min; *p* < 0.017) and the subsequent steady-state phase (*p* < 0.017). Crucially, no statistically significant difference was identified between the legacy NAV-TKA cohort and the steady-state RA-TKA Proficiency phase (Median: 77.00 min; *p* > 0.017), mathematically validating that the surgical team successfully achieved operative time neutrality once the initial interface adaptation was complete. For the non-computer-assisted steps and baseline manual workflow, no statistically significant global differences were observed in Registration time (T_reg_, *p* = 0.945, χ^2^ = 0.11) or Manual Phase time (T_man_, *p* = 0.052, χ^2^ = 5.90), confirming high institutional workflow reproducibility that remained unaffected by the introduction of the robotic effector. Conversely, Planning time (T_plan_) introduced a significant global difference (*p* < 0.001, χ^2^ = 78.39), with post-hoc testing confirming that conventional navigation (Median: 0.75 min) was significantly faster than both the robotic Learning (2.08 min) and Proficiency (2.05 min) phases (*p* < 0.017), representing a mandatory, constant intra-operative time investment of approximately 1.3 min.

**Table 2:** All values are expressed in minutes as Median [25th–75th Percentiles/IQR]. Significance level set at p < 0.05

Phase	NAV-TKA (*n* = 142)	RA-TKA lear. (*n* = 58)	RA-TKA prof. (*n* = 36)	Global *P*-Value^b^ (Kruskal-Wallis)	RA-TKA all^a^ (*n* = 94)
T _reg_	4.48 [3.75–5.35]	4.25 [3.95–5.00]	4.32 [3.97–5.50]	0.945 (χ^2^ = 0.11)	4.27 [3.97–5.11]
T _plan_	**0.75** **[0.27–1.43]**	**2.08** **[1.55–2.55]**	**2.05** **[1.35–2.37]**	< 0.001* (χ^2^ = 78.39)	2.07 [1.50–2.51]
T _cut_	**19.08** **[17.20–22.43]**	**20.30** **[18.10–22.20]**	**17.95** **[16.15–19.58]**	< 0.001* (χ^2^ = 18.08)	19.61 [17.62–21.64]
T _man_	54.27 [50.38–58.35]	56.10 [51.20–61.50]	54.33 [48.88–57.30]	0.052 (χ^2^ = 5.90)	55.60 [50.66–60.87]
T _s2s_	**81.00** **[76.00–86.00]**	**84.00** **[79.00–91.00]**	**77.00** **[74.75–89.00]**	< 0.001* (χ^2^ = 15.10)	82.50 [77.00–89.00]

ª RA-TKA all represents the overall robotic cohort and is reported for descriptive completeness

ᵇ The global p-value is calculated using the Kruskal-Wallis test exclusively across the three mutually exclusive and independent cohorts: NAV-TKA, RA-TKA learning phase and RA-TKA proficiency phase. Post-Hoc Pairwise Comparisons (Dunn’s test with Bonferroni corrected α = 0.017)

For T_plan_, navigation is significantly faster than both the Learning and Proficiency phases (p < 0.017)

For T_cut_, all pairs are significantly different (p < 0.017); the Proficiency phase is significantly faster than both navigation and the Learning phase, while the Learning phase introduces a temporary time penalty compared to navigation

For T_s2s_, the Learning phase is significantly longer than both navigation and the Proficiency phase (p < 0.017), whereas no statistically significant difference exists between navigation and the Proficiency phase (p > 0.017), establishing steady-state time neutrality

### Learning curve and resection efficiency

**Fig. 5:**
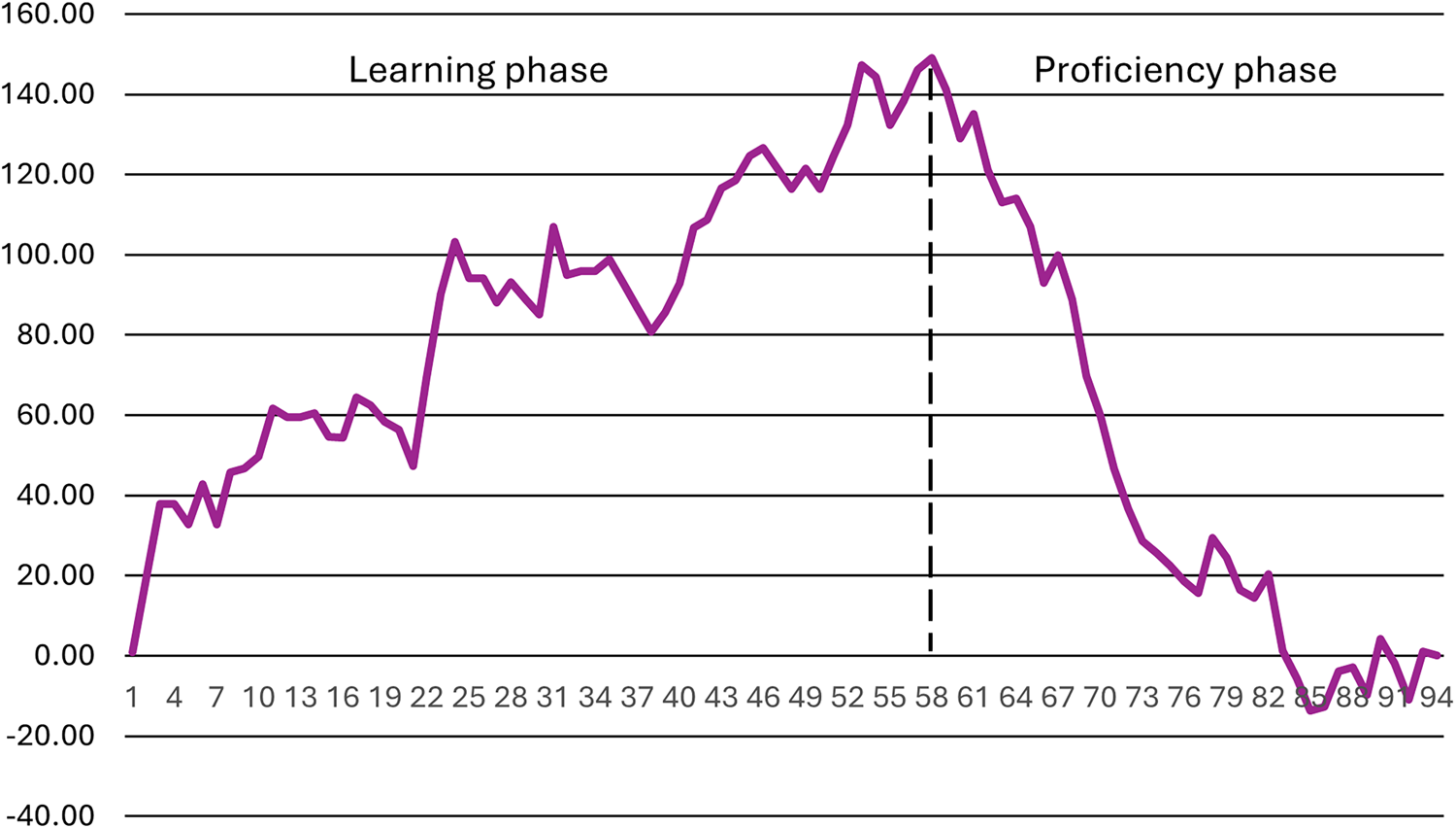
Cumulative Sum (CUSUM) control chart for total skin-to-skin time in the RA-TKA cohort. The curve shows an initial learning phase followed by a clear inflection point at case 58 (vertical line), after which the operative times consistently trend below the cumulative average, indicating the achievement of proficiency

The chronological Skin-to-Skin time CUSUM analysis of the 94 consecutive RA-TKA cases identified a distinct inflection point at case 58, which marks the definitive boundary between the initial Learning Phase and the subsequent steady-state Proficiency Phase (Fig. 5). Inferential subgroup analysis highlighted that the primary operational advantage of the active robotic effector lies within the bone resection phase (T_cut_), which exhibited a significant global variation (*p* < 0.001, χ^2^ = 18.08). Dunn’s post-hoc pairwise test confirmed that all group pairings were distinct (*p* < 0.017): during the initial Learning phase, the introduction of the robot generated a temporary execution penalty, with resection times significantly longer (20.30 min) than manual navigation (19.08 min; *p* < 0.017).However, upon entering the Proficiency phase, active robotic positioning accelerated significantly, achieving a median resection time of 17.95 min, which was statistically faster than both the initial learning experience (*p* < 0.017) and the optimized manual navigated cutting technique (19.08 min; *p* = 0.009). This significant execution gain of approximately 1.1 min in the bone removal phase effectively offset the mandatory 1.3-minute planning time penalty (T_plan_), acting as the primary surgical driver for the observed total skin-to-skin steady-state neutrality.

## Discussion

The introduction of robotic assistance in Total Knee Arthroplasty (TKA) has historically confronted surgeons with a difficult trade-off: gaining execution precision at the cost of operative efficiency [10, 11]. The present study confirmed our primary hypothesis, demonstrating that the integration of an imageless open-platform robotic system (ROBIN, Orthokey Italia s.r.l., Italy) into an established navigation workflow does not significantly increase total operative duration compared to the standard navigated technique.

Specifically, the system demonstrated an absence of a statistically significant increase in overall skin-to-skin time since its prospective introduction, avoiding the massive initial procedural delays routinely reported in early robotic experiences. Furthermore, once the initial learning curve was successfully overcome, the active robotic effector demonstrated a statistically significant superiority in bone resection speed.

These findings challenge the prevailing narrative that robotic surgery inevitably compromises operating room throughput and efficiency [12, 13]. The impact of robotics on surgical flow is a critical economic and logistical concern. A recent meta-analysis reported that robotic procedures were, on average, nearly 10 min longer than navigated ones [12]. Similarly, comparisons in high-volume centers have shown that robotic assistance can add approximately 8 min to the procedure time and over 30 min to the total room time [13]. In stark contrast, our comparative analysis revealed no significant global inflation in total skin-to-skin time between the baseline navigated cohort and the overall robotic cohort (Median 81.0 vs. 82.5 min).

This high efficiency is primarily attributable to the fact that the primary operator was familiar with the underlying navigated workflow and software architecture from the legacy device. This significantly minimized the cognitive load and software management friction described in other learning curve studies [20, 21], allowing the surgical team to focus exclusively on robotic execution. Our chronological CUSUM analysis identified a learning curve of 58 cases to reach the steady-state proficiency phase. This is consistent with single-center studies utilizing closed proprietary platforms, where proficiency was reached at 62 cases [15], 30–40 cases [16], and 52 cases [17] (Table 3). However, the key differentiator in our study lies in the magnitude of the learning curve effect. The median operative time reduction between our Learning and Proficiency phases was only 7 min. This delta is substantially lower than the performance drops reported in the literature, which range from 9.5 to 35.1 min [14, 16, 17, 19]. This minimal time differential confirms our workflow retention hypothesis: because the team was already optimized on the shared navigation platform, they did not suffer from initial inefficiencies. Recently, Druel et al. (2025) [22] reported a rapid 11-case learning curve using a closed robotic system; however, their mean operative time in the proficiency phase remained at 108 min, substantially longer than our proficiency median of 77.0 min. This underscores that a rapid CUSUM inflection point may reflect initial user adaptation rather than peak absolute operational efficiency.

**Table 3:** Improvement Magnitude Comparison from different authors, and improvement phase begin

Study	System	Cases to proficiency	Time reduction (min)
Current Study	ROBIN	58	7.0
Pagan et al. (2025) [17]	VELYS	52	9.5
Putzer et al. (2024) [19]	MAKO	15	10.0
Neira et al. (2024) [15]	ROSA	62	17.5
Cacciola et al. (2022) [14]	Various	15–40	23.9
Ejnisman et al. (2024) [16]	MAKO/ROSA	30–40	35.1
Druel et al. (2025) [22]	ROSA	11–38	N/A

To ensure that these efficiency findings are valid and not artificially biased by patient selection or procedural variations, strict methodological controls were enforced. First, potential selection bias was mitigated by the extreme homogeneity of the cohorts: all cases involved mild-to-moderate constitutional deformities managed strictly with Cruciate-Retaining (CR) implants (UOC U2 CR), while patients with severe extra-articular malalignments or fixed flexion contractures > 15° were systematically excluded. Second, a single senior surgeon (Y.V.) performed all procedures utilizing an identical lateral approach (100% of cases) and a strict Functional Alignment philosophy. Rather than pursuing an unyielding mechanical neutrality, bone cuts were dynamically adjusted in real-time based on the patient’s native soft-tissue laxity, minimizing time-consuming ligamentous releases in both cohorts. Finally, the operating room staff (first assistant and scrub nurse) was standardized and dedicated. This rigorous protocol guarantees that the observed operational parity is an intrinsic characteristic of the platform’s interface design rather than an artifact of a clinical learning curve or changing surgical strategies.

Furthermore, a distinction in our design was the strict exclusion of cases performed in the presence of external visiting surgeons (*n* = 20). As noted by Pagan et al. (2025) [17], educational disruptions and intra-operative teaching pauses in the OR can artificially inflate operative times. By removing these confounding cases, we isolated the intrinsic technical performance of the workflow, revealing that even during the initial Learning Phase, robotic resection times remained controlled.

A novel finding of this study is the remarkable efficiency of the active robotic effector once proficiency is attained. In the steady-state phase, the robotic resection time (T_cut_) became significantly faster than manual navigated resection (17.95 vs. 19.08 min; *p* = 0.009). This directly contradicts the common perception that active robotic sawing is inherently slower than manual cutting due to rigid software boundaries and haptic constraints. This substantial execution gain of approximately 1.1 min during bone removal effectively offsets the time investment in the planning phase (T_plan_: +1.3 min), acting as the primary mathematical driver for the total skin-to-skin parity. This aligns with recent assertions by Ho et al. (2025) [20] suggesting that optimized, integrated robotic workflows can actively reduce computer-assisted working times compared to legacy configurations.

From an institutional and economic perspective, demonstrating that a robotic system imposes no sustained time penalty over an optimized navigation benchmark suggests a highly favorable cost-effectiveness profile. Previous health economic analyses noted that the potential clinical savings of robotics (reduced length of stay, fewer outliers) are frequently eroded by increased theater occupancy costs and reduced surgical throughput [4, 10, 11]. Our data suggest that public high-volume centers can seamlessly adopt open-platform robotic precision to access its documented radiographic and clinical benefits without compromising surgical volume or incurring unsustainable overtime staffing costs.

This study has limitations that must be acknowledged. First, it is a retrospective, single-center analysis, which may restrict the immediate generalizability of the absolute durations. Second, while the ROBIN and BLU-IGS systems reasonably represent their respective technological categories in this study, they cannot fully capture the entire spectrum of RA-TKA and NAV-TKA architectures available on the market, which may differ substantially across proprietary interfaces and surgical workflows. Third, because the control group (NAV-TKA) was experienced and optimized, it presented an extremely high baseline benchmark for comparison. Fourth, we did not analyze radiographic outcomes or clinical PROMs in this manuscript, as the primary objective was strictly limited to workflow segmentation and operational efficiency. Finally, from a statistical standpoint, our retrospective design evaluated superiority; since it lacked a predefined equivalence or non-inferiority margin, our conclusions remain descriptive regarding workflow parity and must not be interpreted as a formal statistical proof of equivalence. Future prospective studies will evaluate whether this operational efficiency translates into superior long-term functional outcomes.

## Conclusion

The results of this study challenge the widely held assumption that incorporating robotic precision into knee arthroplasty inevitably compromises surgical efficiency. We demonstrated that integrating an open-platform robotic system (ROBIN) into an established navigation workflow (BLU-IGS) allows for the seamless adoption of robotic assistance with no statistically significant increase in total skin-to-skin operative times compared to legacy computer-navigated techniques. Although a clear learning curve of 58 cases was mathematically identified, the minimal operative time delta between the learning and proficiency phases (7 min) demonstrates that retaining a familiar software interface effectively cushions the initial efficiency loss typically associated with closed-platform robotic systems.

Crucially, our findings highlight a specific performance advantage: upon achieving proficiency, active robotic execution performs bone resections significantly faster than manual navigated instrumentation (17.95 vs. 19.08 min; *p* = 0.009). This technical execution gain effectively counterbalances the mandatory intra-operative planning time investment. By eliminating the historical “time penalty” barrier, this integrated workflow model offers a logistically sustainable pathway to implement high-precision surgery without disrupting operating room throughput or compromising institutional healthcare economics.

## Data Availability

The datasets generated during and/or analyzed during the current study are available from the corresponding author on reasonable request.
